# Colloidal cholesteric liquid crystal in spherical confinement

**DOI:** 10.1038/ncomms12520

**Published:** 2016-08-26

**Authors:** Yunfeng Li, Jeffrey Jun-Yan Suen, Elisabeth Prince, Egor M. Larin, Anna Klinkova, Héloïse Thérien-Aubin, Shoujun Zhu, Bai Yang, Amr S. Helmy, Oleg D. Lavrentovich, Eugenia Kumacheva

**Affiliations:** 1 Department of Chemistry, University of Toronto, Toronto, Ontario, Canada M5S 3H6; 2 State Key Laboratory of Supramolecular Structure and Materials, College of Chemistry, Jilin University, Changchun 130012, China; 3 The Edward S. Rogers Sr. Department of Electrical and Computer Engineering and the Institute of Optical Sciences, University of Toronto, Toronto, Ontario, Canada M5S 3G4; 4 Liquid Crystal Institute and Chemical Physics Interdisciplinary Program, Kent State University, Kent, Ohio 44242, USA; 5 Institute of Biomaterials & Biomedical Engineering, University of Toronto, 164 College Street, Toronto, Ontario, Canada M5S 3G9; 6 Department of Chemical Engineering and Applied Chemistry, University of Toronto, 200 College Street, Toronto, Ontario, Canada M5S 3E5

## Abstract

The organization of nanoparticles in constrained geometries is an area of fundamental and practical importance. Spherical confinement of nanocolloids leads to new modes of packing, self-assembly, phase separation and relaxation of colloidal liquids; however, it remains an unexplored area of research for colloidal liquid crystals. Here we report the organization of cholesteric liquid crystal formed by nanorods in spherical droplets. For cholesteric suspensions of cellulose nanocrystals, with progressive confinement, we observe phase separation into a micrometer-size isotropic droplet core and a cholesteric shell formed by concentric nanocrystal layers. Further confinement results in a transition to a bipolar planar cholesteric morphology. The distribution of polymer, metal, carbon or metal oxide nanoparticles in the droplets is governed by the nanoparticle size and yields cholesteric droplets exhibiting fluorescence, plasmonic properties and magnetic actuation. This work advances our understanding of how the interplay of order, confinement and topological defects affects the morphology of soft matter.

## Abstract (web summary)

Confinement-induced organization of nanoparticles is utilized to generate new structures with novel optical or magnetic properties. Here, Li *et al.* show that colloidal liquid crystals formed by rod-like nanoparticles self-assemble into various geometries depending on the degree of spherical confinement in droplets.

## Graphical abstract

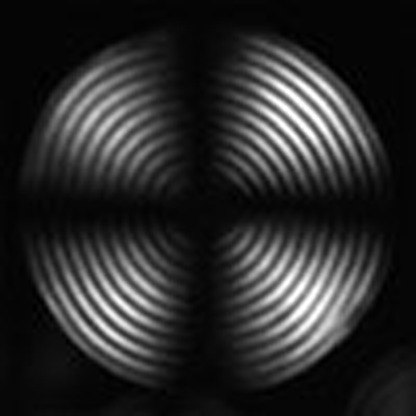

Programmable organization of nanoparticles (NPs) in new structures is an important target of materials science and nanotechnology^1^,^2^,^3^. The organization of NPs in constrained geometries such as thin films or narrow channels is a valuable strategy for the generation of novel structures with potentially useful properties^4^,^5^,^6^,^7^,^8^. Spatial constraints can break the symmetry of confined structures and lead to a strong deviation from an equilibrium morphology. Confinement-mediated organization of particles in spherical space, e.g., in spherical droplets is of particular interest, since it offers the conditions of an isotropic confinement and controlled curvature for fundamental studies of particle packing^9^, self-assembly^10^,^11^ and relaxation of colloidal liquids^12^. Practically, phase separation of colloidal dispersions in spherical confinement can be used for the generation of multicomponent particles, for example, Janus particles^13^. Self-assembly of colloids in droplets paves the way for producing patchy structures mimicking atoms and molecules^14^.

Spherical confinement of liquid crystals formed by NPs offers a large parameter space for the generation of new structures. Macroscopic liquid crystals formed by viruses^15^,^16^ or carbon nanotubes^17^,^18^ have been reported; however, the organization of colloidal liquid crystals in spherical droplets remains a largely unexplored area of research, especially, for cholesteric (Ch) phases. Droplets of colloidal Ch liquid crystals can exhibit topological defects that can be used for fundamental studies of soft matter^19^,^20^ and as templates for the organization of molecules^21^ and NPs^22^,^23^. In comparison with molecular mesogens, the organization of NPs in liquid crystalline droplets adds a new set of length scales, thus making these droplets act as hosts for other types of NPs. Moreover, since the interplay of elastic and surface energies in droplets of thermotropic liquid crystals and the coupling of the properties of liquid crystals and NPs may yield structures with new optical and photonic properties^24^,^25^,^26^, these studies can be extended to colloidal lyotropic systems, especially, if their birefringence is enhanced by additives^6^, or by increasing NP volume fraction^27^.

Here we report the organization of rod-shape cellulose nanocrystals (CNCs) in Ch liquid crystals in the spherical confinement of micrometer-size droplets. In marked difference with droplets formed by thermotropic Ch mesogens^28^, confinement of the Ch–CNC liquid crystal results in phase separation into an isotropic phase in the droplet core and a Ch phase with concentric packing of the CNC layers at the droplet periphery. Further progressive confinement causes a transition to a bipolar planar Ch structure. For the droplets used as hosts for polymer, metal, carbon and metal oxide nanocolloids, we show the capability to control NP partition in the isotropic core and Ch shell and form Ch–CNC droplets with plasmonic, fluorescence and magnetic properties, respectively.

## Results

### Spherical droplets of Ch colloidal liquid crystal

**Figure 1:**
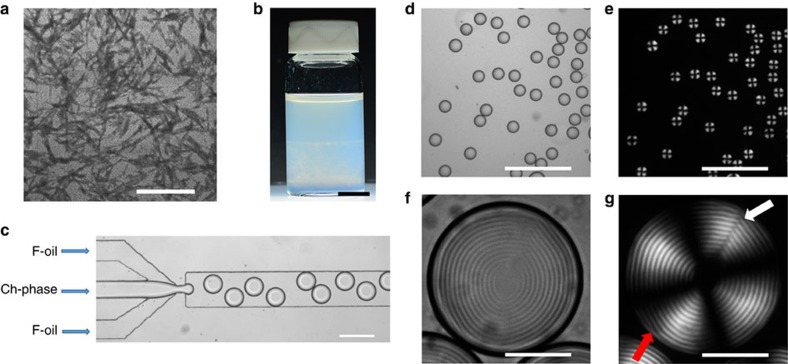
Generation of droplets by microfluidic emulsification of the Ch–CNC suspension. (**a**) TEM images of dried CNCs. The scale bar is 500 nm. (**b**) Phase separation of the macroscopic CNC suspension into a Ch phase (bottom) and an isotropic phase (top). The scale bar is 1 cm. (**c**) Optical microscopy image of microfluidic generation of droplets of the Ch–CNC phase. The flow rate of F-oil and the Ch–CNC phase were 0.8 and 0.2 ml h^−1^, respectively. The scale bar is 250 μm. (**d**,**f**) Bright-field (BF) optical microscopy images and (**e**,**g**) corresponding polarized optical microscopy (POM) images of Ch-CNC droplets with *φ*_0_=0.048. Scale bars are 500 μm (**d**,**e**) and 50 μm (**f**,**g**). Arrows in **g** show the radial disclination line defect running perpendicularly to the Ch layers (white arrow) and an edge dislocation running parallel to the Ch layers (red arrow).

Figure 1a shows a transmission electron microscopy (TEM) image of negatively charged, rod-like CNCs with an average length *L* and diameter *D* of 183 and 23 nm, respectively. An aqueous 7 wt% CNC suspension, with a volume fraction of CNCs of 0.046, was equilibrated for 21 days. Following equilibration, the suspension phase separated into two co-existing phases (Fig. 1b), in agreement with earlier reports^29^,^30^, that is, into an isotropic top phase with the volume fraction of CNCs of 0.044 and a Ch bottom phase with the volume CNC fraction *φ*_0_=0.048. The volume fraction of the Ch phase in this two-phase system was 0.55 (Supplementary Fig. 1a). In the Ch phase, the CNCs exhibited long-range orientational order combined with their helical alignment. Polarized optical microscopy (POM) images of the Ch phase showed a multidomain mosaic pattern with characteristic stripes, with the axis of the Ch helicoid distorted in space (Supplementary Fig. 1b,c). The pitch of the Ch phase measured as a double distance between two adjacent stripes was *P*=6.0±0.3 μm.

The Ch phase was separated from the two-phase system and emulsified in a microfluidic flow-focusing droplet generator^31^. A fluorinated oil (F-oil) containing 1.0 wt% of a surfactant triblock copolymer perfluoropolyether and poly(ethylene oxide-*co*-propylene oxide) and the Ch phase were supplied to the microfluidic device as a continuous and droplet phases, respectively (Fig. 1c). In the orifice, the shear stress imposed by the two outer F-oil streams on the inner thread of the Ch phase resulted in a periodic breakup of the aqueous stream into uniformly sized droplets with a polydispersity of 2.5%. The droplet radius, *R*, was varied from 40 to 60 μm by changing the volumetric flow rate of F-oil from 1.5 to 0.3 ml h^−1^ at a constant flow rate of the Ch phase at 0.2 ml h^−1^.

Figure 1d–g shows the bright-field (BF) and the POM images of the droplets with a radius *R*=45 μm. The POM images showed the characteristic features of the Ch phase, that is, a Maltese cross and a shell with alternating bright and dark concentric rings corresponding to the surfaces of constant refractive index^26^ (a similar ring pattern is shown in the BF image in Fig. 1f). This structure of the droplet shell corresponded to the tangential CNC alignment at the droplet/F-oil interface with a radial orientation of the helical axis 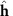 showing the direction along which the local orientation of the CNCs twists in space. The local orientation of the CNCs was described by the so-called director field 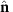. (Note that 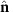 and 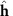 were orthogonal to each other). The average pitch *P* in the Ch–CNC droplets, measured as a double distance between two adjacent concentric rings, was 6.1±0.3 μm, that is, close to *P*=6.0±0.3 μm of the macroscopic Ch phase. The central region of the droplets with a radius of 8.5±1.1 μm was formed by the isotropic phase. The equilibrium droplet structure was reached via transient states^32^. Spherical concentric packing of the Ch layers started at the droplet/F-oil interface, being assisted by the tangential surface anchoring of the CNCs, and with equilibration time, it gradually propagated toward the droplet centre (Supplementary Fig. 2).

Usually, confinement of rods between parallel planar substrates is expected to enhance the orientational order of the system^33^,^34^; however, spherical confinement of Ch phases leads to a different situation, in which the equilibrium state must be accompanied by a particular minimum number of defects, as dictated by topology. A Ch droplet with an infinitely strong tangential anchoring of the director at the droplet surface should acquire spherical packing of Ch pseudo-layers, which implies that the normal 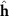 to these layers forms a radial point-defect hedgehog with the core in the droplet centre^32^. In such droplets, the director within each spherical pseudo-layer must contain defects, according to the Euler–Poincaré theorem^32^. When the spherical pseudo-layers are confined in a concentric manner within the Ch droplet, these point defects form radial defects—disclinations—in the local director field 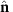 (ref. 32). Furthermore, if the equilibrium is not perfect, the Ch droplet may contain additional defects, such as dislocations that are associated with, for example, partial insertion or removal of the concentric layers.

Thus the equilibrium state of the Ch–CNC droplets with a tangential alignment of the director at the Ch/F-oil interface could not be realised without a point defect in the radial distribution of the normal 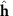, accompanied by the radial disclination lines in the director field 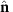. Near the equilibrium, dislocation defects were also possible.

The experimental results, in general, supported these expectations. In Fig. 1f,g, the point defect is evidenced by the concentric packing of the Ch–CNC pseudo-layers (which implies a radial configuration of the field 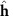). The equilibrium disclination is marked with a white arrow, while an occasionally encountered dislocation is marked with a red arrow. The equilibrium disclinations were necessitated by the spherical confinement topology in the regime of strong chirality, when *R*>>*P*^35^,^36^. As demonstrated in the next section, this ideal structure expected on the basis of topological considerations was realised only partially, since the structure of the Ch–CNC droplets exhibited a strong dependence on their size.

### Size dependence of the internal structure of Ch–CNC droplets

The effect of confinement on the droplet morphology was examined as a function of the droplet size for three cases.

Very-large drops with *R*>115 μm exhibited a multidomain Ch structure, similar to the macroscopic Ch–CNC phase (Supplementary Fig. 3). A multidomain morphology of large droplets with the axis of the Ch helicoid distorted in space was a manifestation of a very-long time needed for their complete equilibration. In this regard, the very-large droplets behaved similarly to the macroscopic Ch–CNC phase.

**Figure 2:**
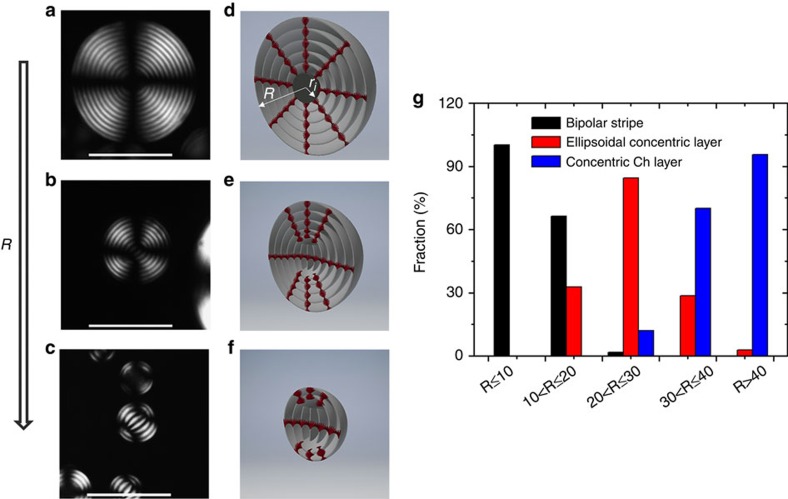
Confinement-mediated structural transitions in Ch-CNC droplets. (**a**–**c**) Representative POM images of the Ch–CNC droplets with dimensions in the range of 40≤*R*≤115 μm (**a**) 10<*R*<40 (**b**) and *R*≤10 μm (**c**). The scale bars are 50 μm. (**d**–**f**) Schematics of droplets shown in **a**,**b** and **c**, respectively. (**g**) Distribution of the populations of droplets with concentric Ch layer, bipolar stripe and ellipsoidal concentric layer structures. *φ*_0_=0.048. For each population, at least, 60 droplets were analysed.

Inspection of droplets with a radius in the range 5≤*R*≤115 μm revealed that they had three types of ‘monodomain' structures (Fig. 2a–c). The corresponding schematics of the CNC orientation in these droplets are shown in Fig. 2d–f, where the Ch layers are presented as surfaces separated by half-pitch. The distribution of the populations of droplets with different morphologies is shown in Fig. 2g.

For large droplets with dimensions in the range 40≤*R*≤115 μm (6.5≤*R/P*≤18.8), ∼96% of the droplets exhibited a Ch shell with a concentric packing of CNC pseudo-layers and thus radial configuration of the normal 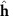 that formed a radial point-defect hedgehog. In these droplets, we observed either one radial disclination of strength 2 (Fig. 1g) or two radial disclinations of strength 1 each (Supplementary Fig. 4). In both cases, the total topological charge was two, equal to the Euler characteristic of the sphere, as required by the Euler–Poincaré theorem^32^. A single radial disclination was observed in ∼90% of the droplets, in agreement with numerical simulations and experimental observations of Ch droplets formed by thermotropic molecular liquid crystals^28^,^37^.

**Figure 3:**
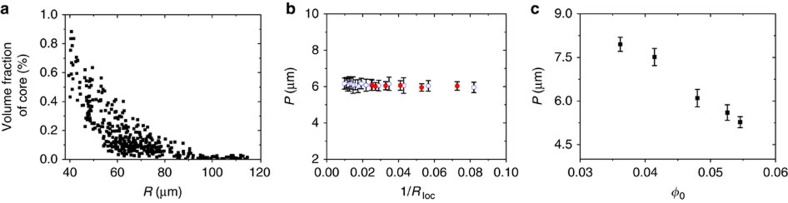
Structural characteristics of droplets with concentric Ch layers. (**a**) Variation in the volume fraction of the isotropic core, plotted as a function of droplet radius *R*, based on the analysis of 390 droplets. (**b**) Pitch variation in the Ch–CNC droplets with a radius of 45 (red spheres) and 100 μm (blue circles), plotted as a function of the local curvature (1/*R*_loc_) of the droplet. *R*_loc_ was measured from POM images as the radial distance between the droplet centre and bright even rings in the Ch shell, starting from the second one. The error bars represent the s.d. For each population of droplets, at least, 10 droplets were analysed. In **a**,**b** *φ*_0_=0.048. (**c**) Pitch variation plotted as a function of CNC volume fraction in the droplets. For each experimental point, at least, 20 droplets were analysed. The error bars represent the s.d.

The most striking feature of the droplets with a concentric Ch layer packing was that the radial point defect exhibited an isotropic core with a radius, *r*_i_, in the range from ∼3 to 8.3 μm (∼0.5–1.4 *P;* Fig. 2a,d). The size of the core was larger in smaller droplets (Supplementary Fig. 5). With the droplet radius *R* decreasing from 115 to 40 μm, the volume fraction ∼*r*_*i*_^3^/*R*^3^ of the isotropic core in the droplet increased from 2 × 10^−5^ to 9 × 10^−3^ (Fig. 3a). This result suggested that spherical confinement caused phase separation in the system that exhibited a single-phase Ch behaviour in macroscopic samples, with the isotropic phase appearing at the core of the radial point defect. It was thus of interest to explore whether the same spherical confinement conditions would cause a change in the Ch pitch in the droplets. Fig. 3b shows the effect of the local curvature 1/*R*_loc_ on the pitch of the Ch shell, where *R*_loc_ was measured from POM images as the radial distance between the droplet centre and bright concentric rings in the Ch shell, starting from the second ring. For droplets with a radius of 45 and 100 μm, filled with the Ch phase at *φ*_0_=0.048, with the local curvature increasing from 0.01 to 0.08, the pitch remained invariant at *P*≈6.1 μm. On the other hand, similarly to the macroscopic Ch–CNC phase^29^, the Ch pitch in the droplets depended on the CNC concentration: when *φ*_0_ increased from 0.036 to 0.054, the pitch changed from 7.9 to 5.2 μm (Fig. 3c).

Intermediate-size droplets with 10≤*R*≤40 μm exhibited a transitional ‘ellipsoidal concentric layer' pattern corresponding to planar Ch pseudo-layers in the centre and tangential CNC orientation at the droplet periphery (Fig. 2b,e). A large fraction of droplets with *R*≤20 μm (*R/P*≤3.3) displayed a stripe pattern characteristic of flattened Ch layers trapped between the two diametrically opposite poles (Fig. 2c). Notably, the interpretation of POM images for the intermediate-size droplets and for the small droplets with *R≤*10 μm (see below) depended on droplet orientation: when the axis of the Ch structure pointed up, a concentric ring structure was observed. Thus the morphology of these droplets was examined by carefully rotating them in the fluid cell (see, for example, Supplementary Movie 1).

**Figure 4:**
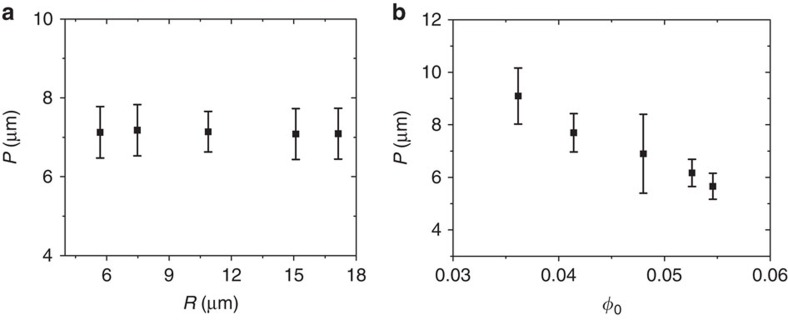
Structural characteristics of the droplets with bipolar patterns. (**a**) Variation in pitch, plotted as a function of droplet radius *R*. *φ*_0_=0.048. (**b**) Pitch variation as a function of CNC volume fraction in the droplets. For each experimental point, at least, 30 droplets were analysed. The error bars represent the s.d.

Small droplets with *R*≤10 μm exhibited almost exclusively a bipolar stripe pattern. For such droplets, at *φ*_0_=0.048, the pitch remained invariant at 7.1 μm with increasing droplet size (Fig. 4a). When the droplets were formed from the Ch–CNC phase with varying *φ*_0_, the pitch showed the dependence on the volume fraction of the CNCs: an increase in *φ*_0_ from 0.036 to 0.054 resulted in the reduction of the pitch from 9.1 to 5.7 μm, respectively (Fig. 4b).

The transition from the ellipsoidal concentric layers to flattened Ch layers occurred at approximately *R*_tr_≈10 μm. For the droplet size of *R*_tr_, the elastic energy of spherical packing of the Ch-CNC layers that scales as *KR*, and the surface anchoring energy of the planar CNC layers that scales as *WR*^2^, were approximately equal to each other^32^. The balance of these two energies enabled the estimation of the surface anchoring strength, *W*, at the Ch droplet/F-oil interface, as *W*≈*K*/*R*_*tr*_≈10^−6^ J m^−2^, where *K* is the average value of the Frank elastic constant that was assumed to be on the order of 10 pN (refs 38, 39), as for other lyotropic liquid crystals.

### Distribution of nanocolloids in the Ch–CNC droplets

In assumption that the micrometer-size isotropic core in the Ch–CNC droplets can be used for the incorporation of chemicals or other nanocolloids, we loaded the droplets with spherical negatively charged 54 and 184 nm-diameter fluorescent dye-labelled polystyrene latex NPs and examined the effect of NP size on their distribution within the droplets. In equilibrium, a macroscopic mixture of the Ch–CNC phase and latex NPs phase separated into a latex-rich isotropic phase and a CNC-rich Ch phase (Supplementary Fig. 6 and Supplementary Note 1). The volume fraction of the isotropic phase increased at a higher volume fraction of latex NPs, *φ*_NP_.

**Figure 5:**
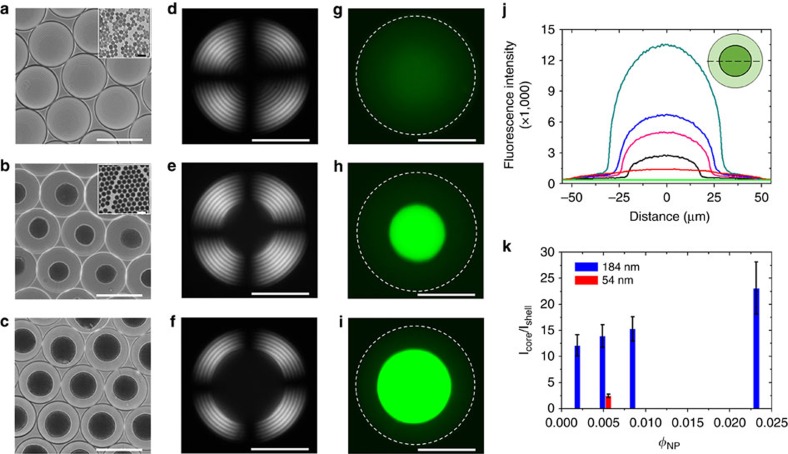
Loading of Ch-CNC droplets with latex NPs. BF (**a**–**c**), POM (**d**–**f**) and FM (**g**–**i**) images of droplets containing fluorescein isothiocyanate dye-labelled 54 nm latex NPs at *φ*_NP_=0.0052 (**a**,**d**,**g**) and 184 nm dye-labelled latex NPs at *φ*_NP_ of 0.0052 (**b**,**e**,**h**) and 0.0235 (**c**,**f**,**i**). The scale bars in **a**–**c** are 100 μm; the scale bars in **d**–**i** are 50 μm. Insets in **a**,**b** show the corresponding TEM images of 54 and 184 nm latex NPs. The scale bar in insets is 100 nm in a and 300 nm in **b**. (**j**) Representative fluorescence line profiles of the Ch-CNC droplets loaded with 54 nm latex NPs at *φ*_NP_=0.0052 (red) and 184 nm NPs at *φ*_NP_ of 0 (green), 0.0022 (black), 0.0052 (magenta), 0.0088 (blue) and 0.0235 (dark cyan). Inset shows the schematic of a droplet with a fluorescence line profile drawn through the droplet centre (dashed line). To prepare droplets with *φ*_NP_=0, the Ch-CNC phase was mixed with a supernatant of the latex dispersion. (**k**) Variation in the core-to-shell fluorescence intensity ratio plotted as a function of NP volume fraction in the droplets. In **a**–**k**, *φ*_0_=0.043. For each value of *φ*_NP_, at least, 30 droplets were analysed. The error bars represent the s.d.

**Figure 6:**
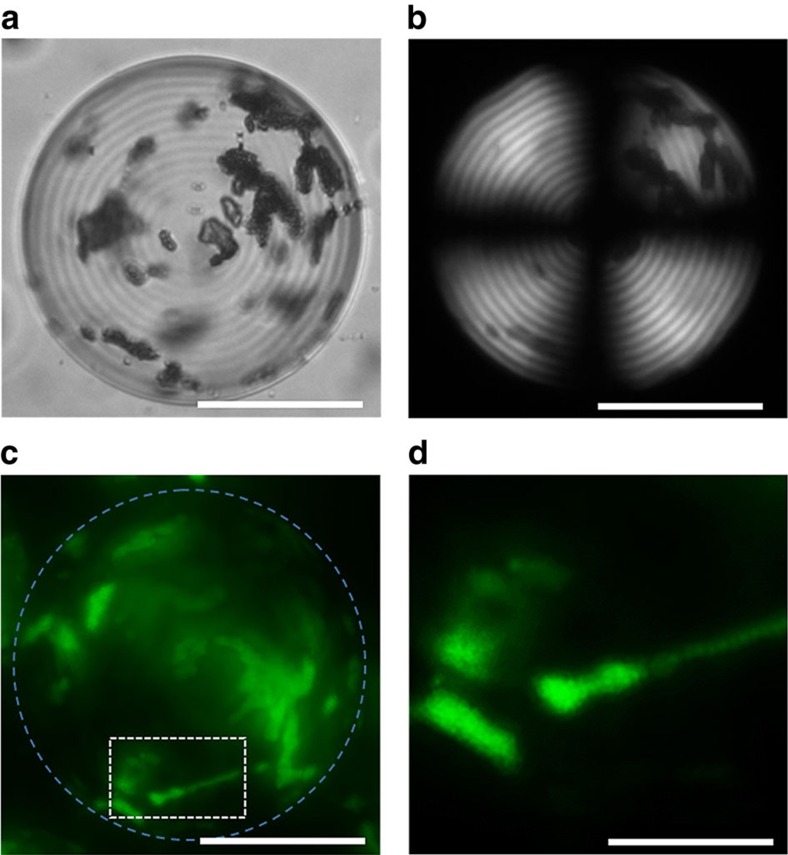
Ch-CNC droplets loaded with 1000, nm latex particles. (**a**) BF, (**b**) POM and (**c**) FM images of aggregates of 1,000 nm latex particles in the Ch-CNC droplets with *φ*_0_=0.043. *φ*_NP_=0.0052. (**d**) Enlarged FM image of the latex particle aggregates shown in the white box in **c**. The scale bar is 50 μm in **a**–**c** and 15 μm in **d**.

Droplets were generated in the microfluidic droplet generator immediately after mixing the Ch phase and latex NPs at *φ*_NP_=0.0052 and subsequently, equilibrating droplets for 72 h. In the droplets, the NPs segregated in the cores of disclinations, similarly to the effect described for disclinations in thermotropic systems^21^,^40^,^41^ and in the isotropic core of the radial hedgehog (Supplementary Figs 7 and 8, respectively). The Ch shell structure of the droplets with a pitch *P* of ∼7 μm was preserved. The extent of partition in the droplet core increased with NP dimensions (Fig. 5a–h and Supplementary Fig. 9a–d). Examination of the variation of the ratio of the core-to-shell fluorescence intensities, based on the fluorescence microscopy (FM) images of the droplets revealed that ∼70 and ∼93% of 54 and 184 nm NPs, respectively, resided in the isotropic core of the hedgehog defect (Fig. 5j,k). Because of the stronger phase separation between the CNCs and the latex NPs with increasing latex content in the system, the extent of latex segregation in the isotropic core increased at higher *φ*_NP_ (Fig. 5k and Supplementary Figs 10–12), and the radius of the isotropic core increased from 12 to 30 μm (Fig. 5j and Supplementary Fig. 11). In contrast with small latex NPs, larger microspheres with a diameter of 1,000 nm formed multi-particle aggregates randomly distributed within the droplets (Fig. 6 and Supplementary Fig. 9e,f).

**Figure 7:**
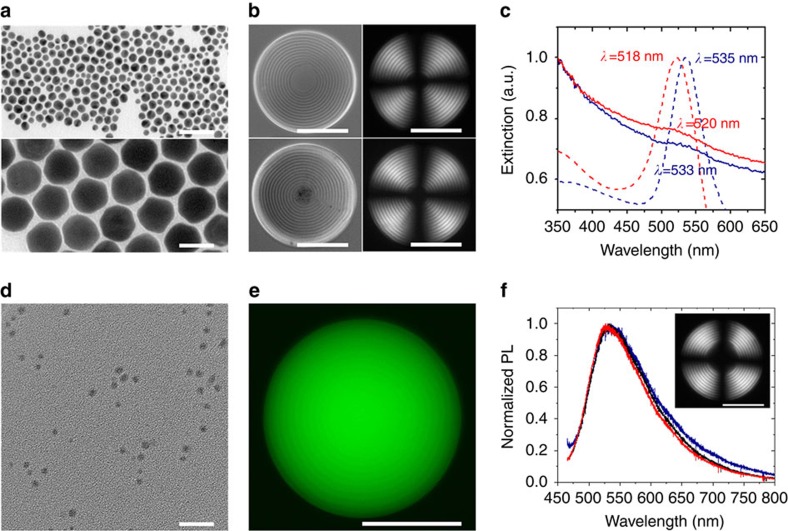
Co-assembly of CNCs and inorganic nanoparticles. (**a**) TEM images of 10 nm (top) and 50 nm (bottom) gold NPs. The scale bar is 50 nm. (**b**) BF (left panel) and POM (right panel) images of the Ch-CNC droplets loaded with 10 nm gold NPs (top panel) and 50 nm gold NPs (bottom panel). The concentration of gold NPs in the droplets is 0.1 mg ml^−1^. The scale bar is 50 μm. (**c**) Extinction spectra of aqueous gold NP dispersion (dashed lines) and Ch-CNC droplets (solid lines) loaded with 10 nm (red lines) and 50 nm (blue lines) gold NPs. The broadening of extinction spectra of the NP-loaded droplets was caused by the light scattering by droplets in the visible light region. (**d**) TEM images of carbon dots. The scale bar is 20 nm. (**e**) FM image of the Ch-CNC droplet carrying carbon dots at concentration of 1 mg ml^−1^. The scale bar is 50 μm. (**f**) Photoluminescence spectra of the core (black line) and shell (red line) of the Ch-CNC droplets loaded with carbon dots, and an aqueous dispersion of carbon dots (blue line), both excited at *λ*_exc_=440 nm. Inset: POM image of the droplet loaded with carbon dots. The scale bar is 50 μm. *φ*_0_=0.043 in **b**–**f**.

With the goal to extend the range of functionalities of the Ch–CNC droplets, we examined the organization of plasmonic gold NPs, fluorescent carbon NPs (carbon dots) and magnetic iron oxide rod-shape NPs, in the Ch–CNC droplets. In comparison with molecular Ch liquid crystals, a relatively large (∼30 nm (ref. 29)) average spacing between the CNCs in the Ch–CNC phase could accommodate small-size NPs. Droplets loaded with NPs were generated by the microfluidic emulsification of the Ch phase mixed with NPs. Figure 7a, top and bottom, shows transmission electron microscopy images of 10 and 50 nm gold NPs capped with thiol-terminated poly(ethylene glycol) with a molecular weight *M*_n_=5,000 g mol^−1^. Small 10 nm gold NPs exhibited a uniform spatial distribution (Fig. 7b, top) in the core-shell Ch–CNC droplets, while 50 nm NPs partitioned in the isotropic droplet centre (Fig. 7b, bottom). Extinction spectra of the droplets loaded with gold NPs showed surface plasmon resonance a wavelengths similar to the corresponding bands of NPs in a macroscopic aqueous dispersion (Fig. 7c), indicating good NP dispersibility in the droplets and the capability to form plasmonic Ch–CNC droplets, with the NPs residing either in the Ch phase, or in the isotropic phase.

Droplets loaded with ∼3.5 nm-size fluorescent carbon dots (Fig. 7d) exhibited a uniform distribution in the Ch–CNC droplets, based on the distribution of fluorescence intensity within the droplets (Fig. 7e and Supplementary Fig. 13). The droplets exhibited a green emission colour when the NPs were excited at *λ*_exc_=440 nm (Fig. 7f), while blue and red emission colours were obtained at *λ*_exc_ of 365 and 550 nm, respectively (Supplementary Fig. 14), due to the intrinsic excitation-dependent properties of carbon dots^42^.

**Figure 8:**
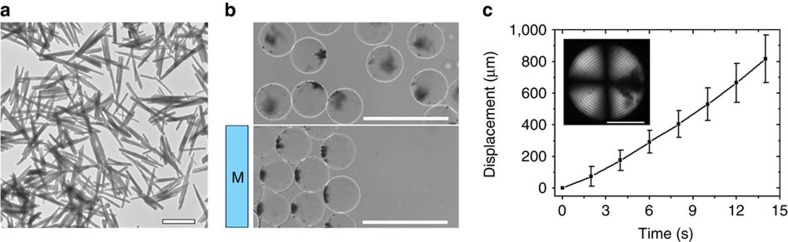
Magnetic actuation of Ch-CNC droplets loaded with magnetic nanorods. (**a**) TEM images of magnetic Fe_3_O_4_/SiO_2_ rods. The scale bar is 1,000 nm. (**b**) BF images of Ch-CNC droplets loaded with magnetic nanorods at concentration of 1.5 mg ml^−1^ without (top image) and with (bottom image) application of magnetic field. The scale bar is 250 μm. (**c**) Displacement of droplets loaded with Fe_3_O_4_/SiO_2_ rods under magnetic field, plotted as a function of time. Inset: POM image of corresponding droplet. The scale bar is 50 μm. The error bars represent the s.d. *φ*_0_=0.043% in **b**–**c**.

In contrast with small gold NPs and carbon dots, magnetic Fe_3_O_4_/SiO_2_ rods with an average diameter and length of 100 nm and 1 μm, respectively (Fig. 8a), formed micrometer-size aggregates in the droplets (shown in Fig. 8b, top). This effect was consistent with the aggregation of large 1,000 nm latex microspheres in the Ch–CNC droplets. These aggregates moved towards a magnet placed outside the cell filled with the emulsion of the Ch–CNC droplets loaded with magnetic rods (Fig. 8b, bottom). Both translational motion of the droplets with acceleration of 1.7 μm s^−2^ (Fig. 8c and Supplementary Movie 2) and rotational droplet motion (Supplementary Movie 3) were achieved under the action of magnetic field.

## Discussion

The existence of a large isotropic spherical region in the Ch droplet centre (Fig. 2a) was in striking difference with enhanced order in nematic phases confined between planar surfaces^33^,^34^. We ascribe this difference to the spherical confinement of the Ch–CNC phase, which in equilibrium resulted in defects such as linear disclinations and the radial point-defect hedgehog. In contrast with a spherical confinement, an ideal flat confinement in equilibrium would not require any defects, nor isotropic inclusions, since the Ch phase separated at the binodal from its isotropic counterpart should reach a homogeneous structure at equilibrium.

Confinement by the spherical Ch/F-oil interface imposed spherical curvature of the Ch pseudo-layers, due to the finite surface anchoring that sets the helical axis 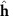 perpendicular to the interface. To realign 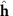 into a tangential orientation, one would need to perform the work against the anchoring energy that scales as *WR*^2^ (ref. 32). On the other hand, the energy of elastic distortions associated with the spherical curvature of pseudo-layers within the droplet scales as *KR*. Therefore, droplets with a large radius, *R*>*K*/*W*, would tend to preserve concentric spherical packing of the Ch pseudo-layers, while satisfying the boundary anchoring conditions, with 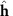 being orthogonal to the Ch/F–oil interface. For *R*>*K*/*W*, the anchoring energy penalty is significantly larger than the elastic cost of the concentric packing, that is, *WR*^2^>>*KR*. Thus for the surface anchoring energy *W*=10^−6^ J m^−2^ and the elastic constant *K*=10 pN, droplets with a radius *R* larger than *R*_tr_=*K*/*W*∼10 μm tend to curve the Ch layers and form concentric spherical layers, to preserve the surface anchoring conditions. On the other hand, when *R*<*K*/*W*, the structure with flattened layers is energetically preferred, that is, *WR*^2^<<*KR*.

The spherical packing of the Ch layers implies a radial configuration of the normal 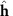 to the Ch layers, with the components 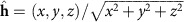 written in the Cartesian coordinates. As one approaches the centre of the droplet at (*x*,*y*,*z*)=(0,0,0), the spatial gradients of 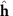 and thus the elastic energy density strongly increase. Such an increase can be avoided if the regions with strong gradients adopt a less ordered structure, by e.g., local melting into an isotropic phase^32^. The effect of isotropic core has been considered in details theoretically for dislinations^43^ and for radial point defects hedgehogs^44^ formed in spherical volumes of a thermotropic nematic liquid crystal doped with a non-mesogenic additive. Analytical solutions are difficult, but the numerical simulations show that the isotropic core of the point defect can have a radius between 2 and 50 nm in pentylcyanobiphenyl (5CB), a typical thermotropic nematic liquid crystal^44^. Recent observations of defect loops with a 10 nm radius formed at the core of point defects in thermotropic liquid crystals support these predictions^45^. In our experiments, we attribute the appearance of a significantly larger core with a radius *r*_*i*_>1 μm, to the lyotropic nature of the liquid crystal system with the low concentration of the solute. Interestingly, large defect cores have been observed in another type of lyotropic liquid crystals, the so-called chromonics^46^.

To understand the mechanism of the appearance of the large isotropic core in the lyotropic Ch–CNC droplets with confinement-imposed spherical packing, we consider the balance of the elastic, condensation and surface energies associated with the cores, following the earlier models^43^,^44^,^46^,^47^. The free energy of the formation of an isotropic core with a radius *r*_i_ in the centre of the Ch droplet with a radius *R*>>*P* and concentric packing of the Ch layers in the droplet shell is


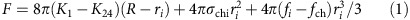


where the first, the second and the third terms are the energy of elastic distortion of the Ch layers in the droplet shell, the surface energy of the Ch-isotropic interface, and the free energy difference between the isotropic and Ch phases, respectively. Here *K*_1_ and *K*_24_ are the splay and saddle-splay elastic constants of the Ch phase, respectively, *σ*_chi_ is the interfacial tension at the Ch-isotropic interface, and *f*_*i*_ and *f*_ch_ are the free energy densities of the isotropic and Ch phases, respectively. In the coarse-grained model^32^ for the gradients of the field 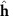 the (volume) free energy density is written as 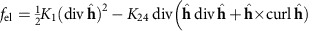.

The first term in equation (1) is obtained by integrating the elastic energy density, *f*_el_ given above, with 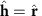 and 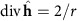, over the Ch–CNC shell volume; here 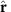 is the radius-vector of the spherical coordinate system, and *r* is the distance from the droplet centre^32^. Since *K*_1_—*K*_24_>0 (to ensure the flat configuration of the Ch layers of an unbounded system)^32^, *σ*_chi_>0 and *f*_i_—*f*_ch_>0, the term that favors the existence of the isotropic core is −8*π*(*K*_1_—*K*_24_)*r*_*i*_, by which the elastic energy is reduced when the highly curved Ch layers in the droplet centre are replaced with an isotropic spherical region. High elastic energy of distortions at the droplet centre is the main mechanism leading to the appearance of the isotropic core.

For a lyotropic system, an isotropic core implies that a fraction of CNCs is transferred from the core into the Ch shell. Therefore, equation (1) should be supplemented by the mass conservation law^44^


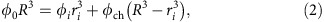


where *φ*_*i*_ and *φ*_ch_ are the CNC volume fractions in the isotropic and Ch regions of the droplet. For the studied range of radii of the Ch–CNC droplets, the volume fraction *φ*_ch_ of the CNC in the Ch shell does not significantly differ from the CNC volume fraction *φ*_0_ of the emulsified single-phase macroscopic Ch–CNC phase. For example, even if all the CNCs were expelled from the isotropic core, for (*r*_*i*_/*R*)^3^ as large as 9 × 10^−3^ (which is the case of droplets with *R*=40 μm) the volume fraction of CNC in the shell, *φ*_ch_=0.0481 would be only a little higher than the initial volume fraction of CNCs *φ*_0_=0.048. The difference *φ*_ch_−*φ*_0_ becomes even smaller as the droplet radius increases. The small difference between *φ*_ch_ and *φ*_0_ explains why the Ch pitch measured in the shells of the biphasic Ch–CNC droplets is almost the same as in the macroscopic Ch phase.

The system of equations (1) and (2) can be solved only numerically^44^, however even a numerical treatment is problematic, since the thermodynamic characteristics of the system under study are not known. To obtain an order of magnitude estimate of the isotropic core radius, we approximate the free energy density difference in equation (1) through the chemical potentials of the isotropic (*μ*_*i*_) and Ch (*μ*_ch_) phases (Supplementary Note 2), as *f*_*i*_−*f*_ch_≈(*μ*_*i*_−*μ*_ch_)/*v*_CNC_, where *v*_CNC_ is the volume of an individual CNC (Supplementary Methods). Furthermore, the difference between the chemical potentials can be estimated on the ground of translational-orientational entropy differences between the isotropic and Ch phases as *μ*_*i*_−*μ*_ch_≈*αk*_*B*_*Tφ*_0_*b*/*v*_CNC_≈*αk*_*B*_*Tφ*_0_*L*/*D*. Here *α* is the numerical parameter of the order of 1, which depends on the exact shape and size distribution of CNCs, *k*_*B*_ is the Boltzmann constant, *T* is the absolute temperature, and *b*=*πL*^2^*D*/4 is the average excluded volume of a CNC with a length *L* and diameter *D*. In what follows, we denote (*μ*_*i*_−*μ*_ch_)/*v*_CNC_≈4*αk*_*B*_*Tφ*_0_/(*πD*^3^) as Δ*f*. To obtain an estimate for the equilibrium radius *r*_i_* of the isotropic core, we assume that the interfacial tension *σ*_chi_ and Δ*f* do not depend on the droplet size. By minimizing the energy in equation (1) with respect to *r*_*i*_, one finds


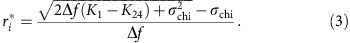


The interfacial tension *σ*_chi_ between the isotropic and Ch phase formed by aqueous CNC suspensions is in the range of 10^−7^−10^−6^ J m^−2^, as determined experimentally^48^. The value of Δ*f* defined above, for *α*=1, is estimated to be 20 J m^−3^. For *K*∼10 pN (refs 38, 39), as for other lyotropic liquid crystals, equation (3) predicts an equilibrium radius *r*_i_* to be on the order of 1 μm, which is close to the experimentally measured core size. Note that for vanishingly small Δ*f*, the equilibrium radius would be *r*_i_*=(*K*_1_−*K*_24_)/*σ*_chi_, thus potentially achieving the value in the range 10–100 μm.

This relatively large, 1 μm or more, core radius is by two or even three orders of magnitude larger than a 10 nm core size of point defects in thermotropic liquid crystals^44^,^45^.The reason for the large core is that the quantity Δ*f* estimated above is significantly smaller than the corresponding latent heat of the isotropic–nematic transition in thermotropic liquid crystals. For typical thermotropic liquid crystals, for example, 5CB, the latent heat is on the order of 10^6^ J m^−3^ (refs 32, 49), that is, five orders of magnitude higher than Δ*f* in the Ch–CNC phase.

The reorganization from concentric to flattened packing of the Ch layers (Fig. 2) observed at decreasing *R*/*P* ratio and caused by the finite surface anchoring at the droplet/F–oil interface was in qualitative agreement with earlier numerical simulations^28^ and experimental results^37^ for thermotropic Ch droplets. We ascribe this structural transition to the balance between the surface energy of the droplets (that scales as *WR*^2^) and the elastic energy of Ch deformations (that scales linearly with the radius, as *KR*)^32^. In large droplets (Fig. 2a), the surface anchoring of CNCs led to concentric packing of strongly curved layers, which remained parallel to the droplet surface, while in small droplets (Fig. 2c), the layers flattened at the expense of partial violation of boundary conditions. Tangential anchoring of the director at the Ch/F–oil interface acted to unwind the Ch helix, thus enlarging the Ch pitch, as observed experimentally.

The collective behaviour of colloid particles in the Ch–CNC droplets was governed by the NP size. Earlier studies of phase separation in mixtures of rods and spheres^16^,^50^,^51^,^52^,^53^, as well as the discussion above on the appearance of the isotropic central core, suggest that phase separation of NPs in the Ch–CNC phase should be expected. The 54 and 184 nm latex NPs fit into the cores of defects and thus gained a freedom of translational motion within an isotropic phase. In the Ch shell, smaller 54 nm NPs were less disruptive to the Ch structure than larger 184 nm NPs, since the average distance between the CNCs is about 30 nm (ref. 29). Placement of 184 nm NPs into a Ch shell was apparently energetically more costly, as these NPs could distort the director. To calculate the actual energy cost of placing the NPs in the isotropic and Ch parts of the droplets more information is needed on surface interactions. The increase in the radius of the latex-rich isotropic cores with increasing *φ*_NP_ stemmed from the stronger phase separation between the latex-rich isotropic phase and the CNC-rich Ch phase, as shown for the macroscopic mixture of the Ch–CNC phase and latex NPs (Supplementary Fig. 6c and Supplementary Note 1).

Larger 1,000 nm-diameter latex beads and 1,000 nm-long magnetic nanorods were observed in both the Ch shells and in the isotropic cores. The plausible reason is that these large particles are kinetically trapped in out-of-equilibrium states during the sample preparation, when the Ch–CNC phase was mixed with the NPs. Large particles perturbed the Ch director around them and attracted each other through long-range elastic forces^50^,^54^,^55^,^56^. If the surface anchoring at the NP–Ch interface is strong, the typical order of magnitude of the elastic energy of interaction is ∼*Kd*, where *d* is the size of the colloid particle. When *d* is 1 μm, the elastic energy of interaction can reach ∼10^−17^ J, that is, orders of magnitude higher than the thermal energy, 4 × 10^−21^ J. Therefore, large particles can form kinetically trapped clusters with irregular shapes that cannot relax into equilibrium shapes, due to very large (compared with thermal energy) energy barriers. A similar behaviour and physical mechanism have been described for the thermotropic Ch phase doped with 1 μm silica particles, which formed irregular clusters and were not responsive to thermal annealing^57^.

Finally, our experiments show that the smallest 3.5 nm carbon dots and 10 nm gold NPs do not exhibit partitioning between different regions of the Ch droplets. These particles could fit between the CNCs and did not perturb the Ch structure.

In conclusion, progressive spherical confinement of Ch phases formed by rod-shape CNC colloids resulted in new structures and transitions between them. Notably, confinement-induced phase separation of the Ch–CNC phase enabled a single-step preparation of core-shell droplets with a controllable Ch shell thickness, a method that is simpler than current methods for the preparation of liquid crystalline shells^58^,^59^. These structures can be used in fundamental studies of defects in frustrated liquid crystalline phases^60^, and for the engineering of particles with unusual mechanical and structural properties^61^,^62^. There is also a potential for optical applications of the droplets, since on evaporation of water from the Ch–CNC phase, the pitch is reduced and the resulting material selectively reflects light in the visible spectral range^30^. In aqueous suspensions, birefringence of CNCs normalized by their volume fraction is ∼0.12 (ref. 27). For the volume fractions of CNCs used in our experiments, the effective birefringence (calculated for quasi-nematic regions of size significantly smaller than the pitch) is rather low, ∼0.006, however, it can be increased by adding special ingredients such as gold nanorods^6^ or by increasing the volume fraction of CNCs^27^.

The co-assembly of CNCs with polymer and inorganic NPs provides a route for the design of new stimulus-responsive liquid crystalline materials. Previously, topological defects have been used as templates for the organization of colloid particles and molecules in thermotropic liquid crystals, which are mostly hydrophobic materials^21^,^41^,^45^,^63^,^64^,^65^,^66^. Our results demonstrate that the topological defect (droplet core) in a hydrophilic medium, such as an aqueous CNC suspension, can also be used for NP organization. The plasmonic, fluorescent and magnetic properties of the NP-loaded Ch–CNC droplets broaden the range of potential applications which remain to be explored.

## Methods

### Generation and characterization of CNC droplets

The experimental details of the synthesis of NPs and the fabrication of microfluidic devices are given in the Supplementary Information (Supplementary Methods). The Ch–CNC droplets were produced by the emulsification of the aqueous Ch–CNC phase in the flow-focusing microfluidic droplet generator^31^. The Ch phase (Supplementary Methods) was injected as a droplet phase at a flow rate of at 0.2 ml h^−1^ into the central channel of the microfluidic device using a syringe pump (PhD 200 Harvard Apparatus PHD 2000 Syringe Pump, USA). The continuous phase (fluorinated oil HFE-7500 or F-oil) mixed with 1 wt% of the copolymer surfactant perfluoropolyether and poly(ethylene oxide-*co*-propylene oxide) was injected into the side channels of the microfluidic device using the second syringe pump (PhD 200 Harvard Apparatus PHD 2000 Syringe Pump, USA) at a flow rate varying in the range from 0.3 to 1.5 ml h^−1^. The Ch–CNC droplets exiting the microfluidic device were collected in a 2 ml glass vial and transferred into a cell consisting of two parallel glass slides separated by a 400 μm-thick spacer. The cell was sealed with epoxy glue and the droplets were equilibrated for various time intervals.

To prepare Ch–CNC droplets with a broad size distribution and radii up to 115 μm, a mixture of 200 μl of the Ch–CNC phase and 1 ml of F-oil was shaken at 50 Hz for 1 min at room temperature in a vortex mixer. For the preparation of droplets with dimensions up to 250 μm, a mixture of 200 μl of Ch–CNC phase and 1 ml F-oil containing 1 wt% of the copolymer surfactant was shaken at 30 Hz for 15 s at room temperature in a vortex mixer. The resultant emulsion of Ch–CNC droplets was introduced in the cell, the cell was sealed with epoxy glue and the droplets were allowed to equilibrate for 7 days.

The BF and POM imaging, and the video recording of the Ch–CNC droplets were carried out on an optical microscope (Olympus BX51) in the transmission mode. The diameters of the droplets and isotropic cores and the pitch of the Ch phase in the droplets were measured using the software ImageJ. The diameter of the isotropic droplet core was defined as the diameter of the innermost circle ring in the droplets, based on the BF images.

### Generation and characterization of droplets loaded with NPs

The Ch–CNC droplets loaded with NPs were prepared by emulsifying a mixture of the Ch–CNC phase and NPs in the flow-focusing microfluidic device. The procedure was identical to that used for the preparation of NP-free Ch–CNC droplets. A mixture of 60 μl of the suspension of FITC-labelled latex NPs, carbon dots, gold NPs or magnetic nanorods and 540 μl of the Ch–CNC phase was prepared by shaking at 50 Hz for 2 min at 25 °C in a vortex mixer and immediately emulsifying this mixture in the microfluidic device.

The hybrid droplets were imaged using an optical microscope (Olympus BX51). The fluorescence images of the Ch–CNC droplets loaded with FITC-labelled latex NPs and carbon dots were acquired on an inverted microscope (Nikon Eclipse-Ti). The fluorescence intensity profiles of these droplets were measured using a software (NIS-Elements AR Analysis). The Z-stack fluorescence images of the Ch–CNC droplets loaded with FITC-labelled latex NPs and carbon dots were taken by Nikon A1 confocal microscope. The extinction spectra of the solution of gold NPs were acquired using Cary-5000 spectrophotometer. The extinction spectra of the emulsion of the Ch–CNC droplets loaded with gold NPs were acquired using a plate reader (CLARIOstar, Mandel) by placing the emulsion of Ch–CNC droplets in a 96-well plate. The photoluminescence of individual Ch–CNC droplets loaded with carbon dots was measured by using a LabRam-HR (JY Horriba). The confocal spectrometer was attached to a microscope to focus on the targeted areas of the droplet. The photoluminescence was excited at *λ*_exc_=444 nm. The videos of translational and rotational movement of the Ch–CNC droplets loaded with magnetic nanorods were taken on an inverted microscope (Nikon Eclipse-Ti). Droplet displacement was measured by a software (NIS-Elements AR Analysis).

### Data availability

The authors declare that the data supporting the findings of this study are available within the article and its Supplementary Information Files, and all relevant data are available from the authors.

## Additional information

**How to cite this article:** Li, Y. *et al.* Colloidal cholesteric liquid crystal in spherical confinement. *Nat. Commun.* 7:12520 doi: 10.1038/ncomms12520 (2016).

## Supplementary Material

Supplementary Information Supplementary Figures 1-14, Supplementary Notes 1-2, Supplementary Methods and Supplementary References

Supplementary Movie 1 A rotational view of droplets with stripe pattern under the cross-polarizer.

Supplementary Movie 2 Translational motion of the droplets under the static magnetic field.

Supplementary Movie 3 Rotational motion of droplet under the rotating magnetic field.
