# Supplementary material for: Colloidal cholesteric liquid crystal in spherical confinement
Source: Nat Commun. 2016 Aug 26;7:12520. doi: 10.1038/ncomms12520 (PMC5007446; doi:10.1038/ncomms12520)
Supplement: Supplementary Information — Supplementary Figures 1-14, Supplementary Notes 1-2, Supplementary Methods and Supplementary References [file ncomms12520-s1.pdf]

Supplementary Figures

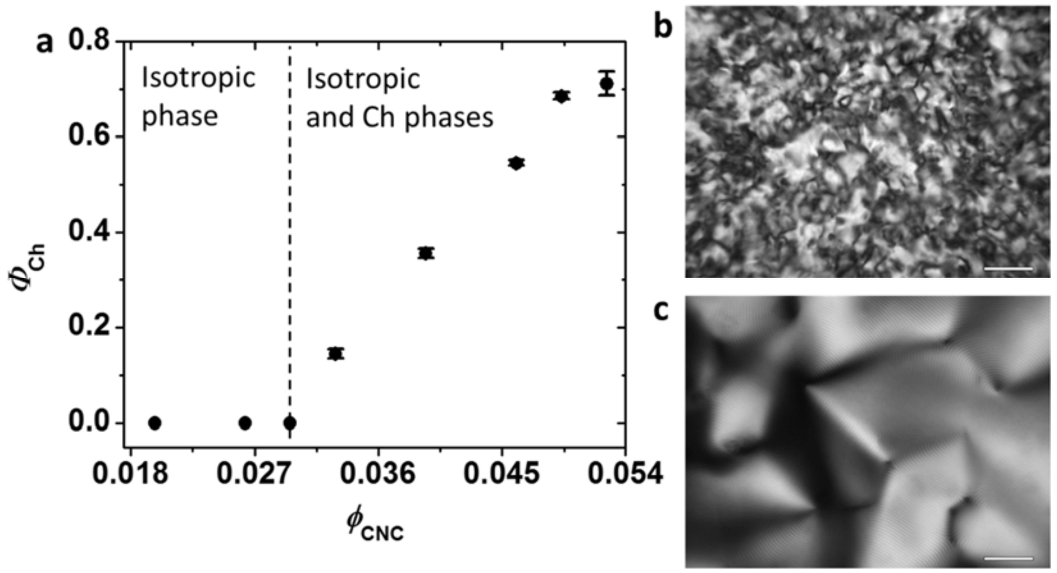

Supplementary Fig. 1. **Phase separation of a macroscopic aqueous suspension of cellulose nanocrystals (CNCs). a,** Variation in the volume fraction of the cholesteric (Ch) phase, plotted as a function of the volume fraction of CNCs in the suspension. **b**, Representative low-magnification polarized optical microscopy (POM) image of the Ch-CNC phase separated from the phase-separated CNC suspension. The scale bar is 200 μm. **c**, High-magnification POM image of the Ch-CNC phase shown in (a). The scale bar is 50 μm. The Ch-CNC phase was stored in a sealed vial for 210 days. ϕ_0=0.048 in b and c.

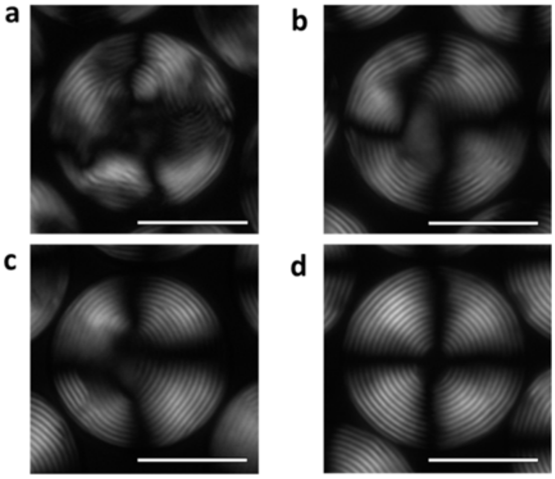

Supplementary Fig. 2. Evolution of the Ch morphology of the Ch-CNC droplets. **a**, POM images taken immediately after the emulsification of the Ch-CNC phase and **b**, 17 h **c**, 41 h (c) and **d**, 72 h after emulsification. The scale bar is 50 μm. ϕ_0=0.048.

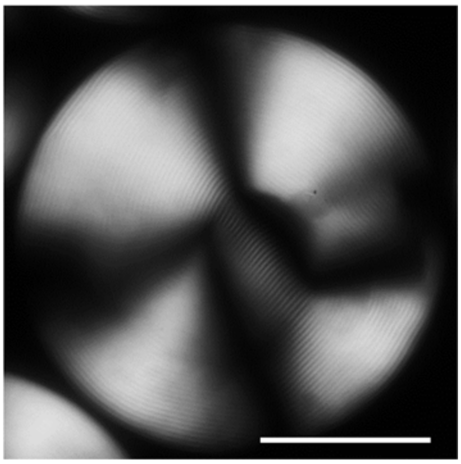

Supplementary Fig. 3. **Multidomain morphology of the Ch-CNC droplets.** POM image of the Ch-CNC droplet with a radius of 130 μm. The scale bar is 100 μm. ϕ_0=0.048.

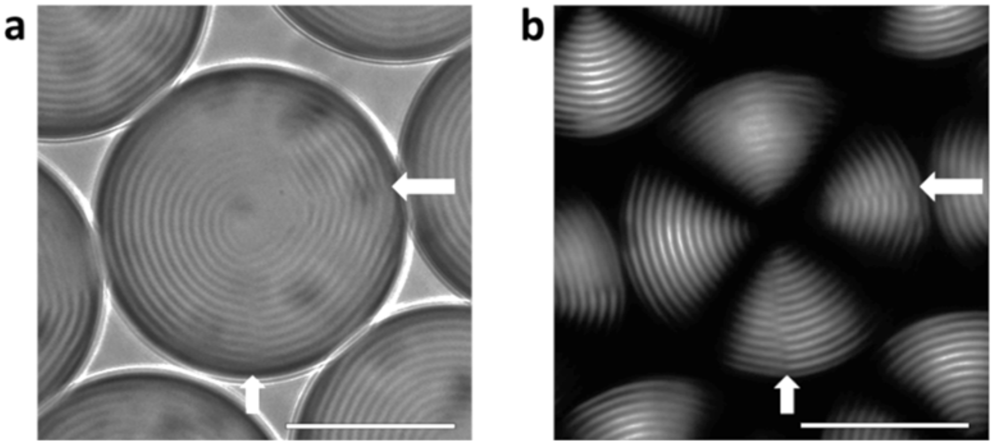

Supplementary Fig. 4. Disclination defects in Ch-CNC droplets. Bright field (BF) (a) and POM (b) images of the Ch-CNC droplet. The arrows show two radial disclination lines of strength 1, each. The scale bar is 50 μm. ϕ_0=0.048.

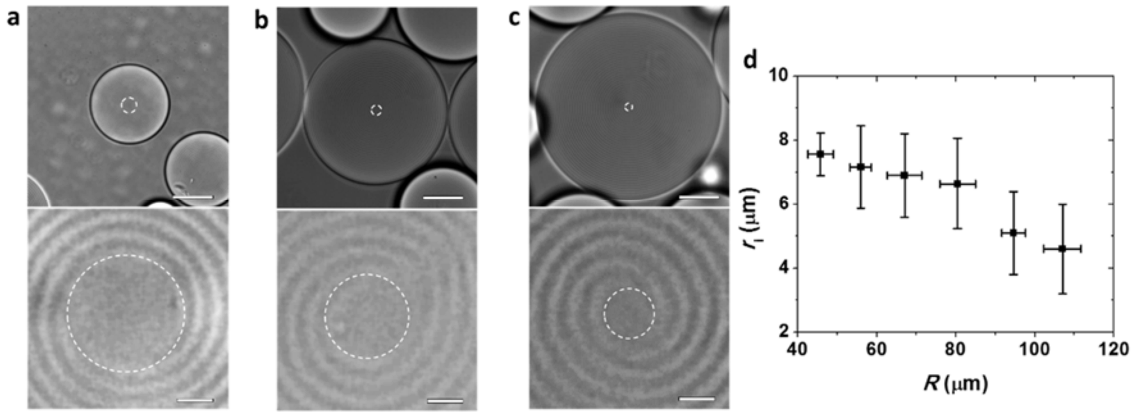

Supplementary Fig. 5. Variation in dimensions of isotropic cores in Ch-CNC droplets. a-c, Low-magnification (top) and corresponding high- magnification (bottom) BF images of the Ch-CNC droplets with *R* of 45 (a), 86 (b), and 111 μm (c). The scale bar is 50 μm (the top row of images), and 5 μm (the bottom row of the images). The isotropic core is surrounded with a dashed white line to assist with visualization. **d**, The variation in the radius of the isotropic core, plotted as a function

of the droplet radius, based on the analysis of 390 droplets. The error bars show standard deviation. ϕ_0=0.048.

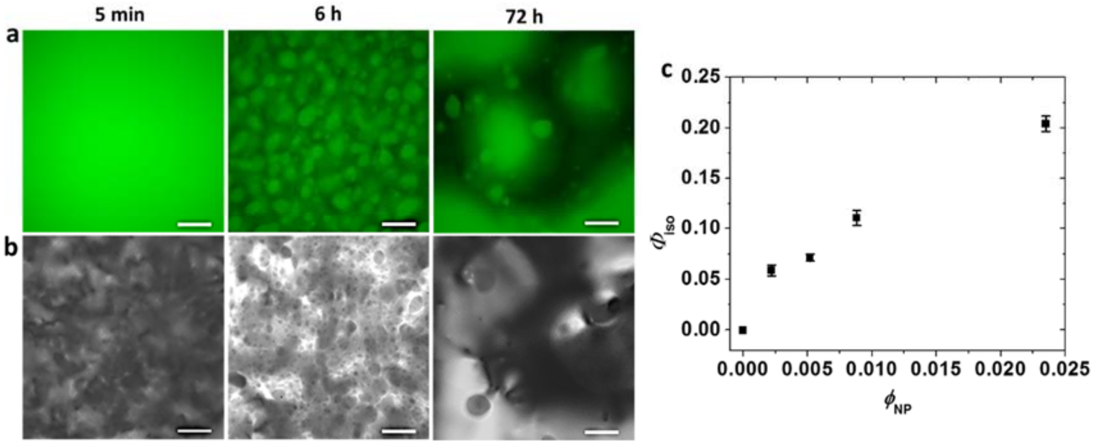

Supplementary Fig. 6. **Phase separation of the macroscopic mixture of the Ch-CNC phase and latex nanoparticles (NPs).** Fluorescence microscopy (FM) (**a**) and POM (**b**) images of the mixture of the Ch-CNC phase and 184 nm latex NPs at ϕ_{NP}=0.0052. The scale bar is 100 μm. The isotropic latex-rich phase appears bright in (a) and dark in (b). The time shown above the images corresponds to the time interval after mixing the Ch-CNC phase and latex NPs. **c**, Variation in the volume fraction of the latex-rich isotropic phase, plotted as a function of latex volume fraction in the suspension of latex NPs and the Ch-CNC phase after equilibration for 18 days. ϕ_0=0.043.

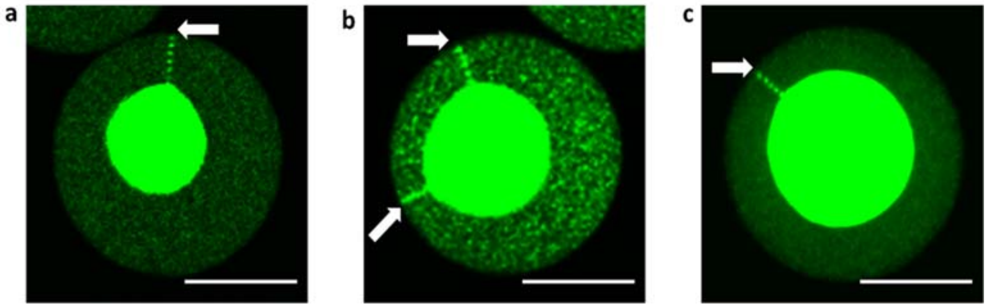

Supplementary Fig. 7. **Partition of latex NPs in disclination defects. a-c**, FM images of the Ch-CNC droplets loaded with 184 nm latex NPs at ϕ_{NP} of 0.0022 (a), 0.0088 (b) and 0.0235 (c). White arrows show the disclination lines. The scale bar is 50 μm. ϕ_0=0.043.

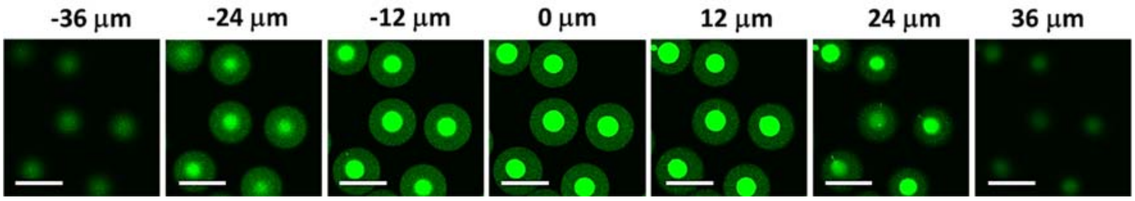

Supplementary Fig. 8. **Z-stack FM images of the Ch-CNC droplets loaded with latex NPs.** The 0 μm position corresponds to the central plane of the droplets, and the images were taken on the top (negative values) to the bottom (positive values) of this plane. The diameter of latex NPs is 184 nm. The scale bar is 100 μm. ϕ_0=0.043. ϕ_{NP}=0.0022.

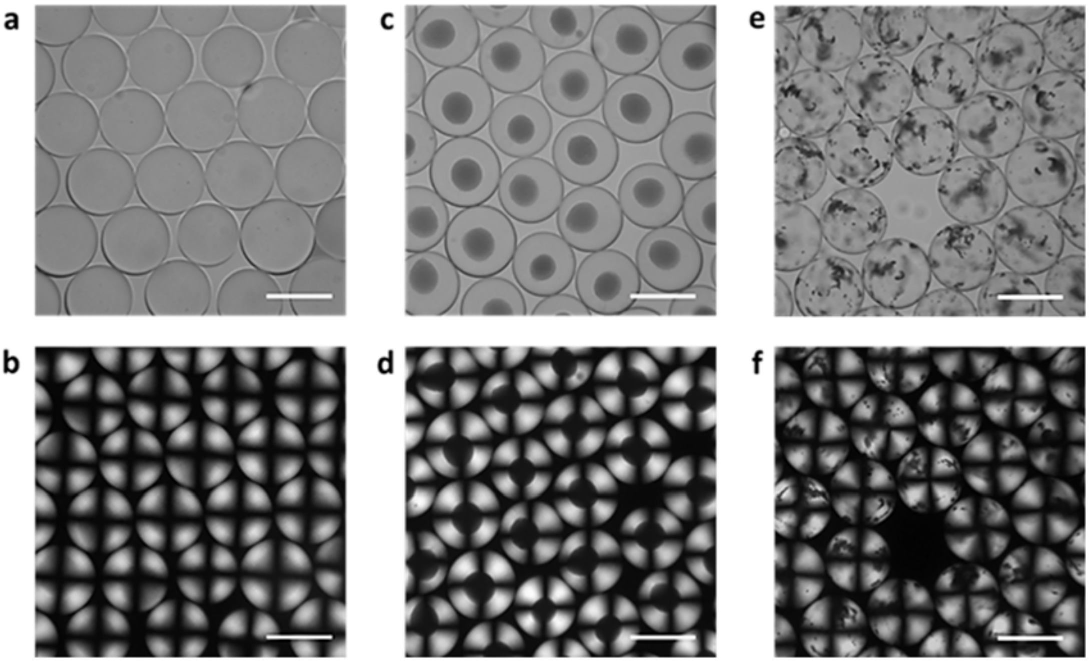

Supplementary Fig. 9. **Low-magnification images of Ch-CNC droplets loaded with different size latex NPs.** BF (a, c and e) and POM (b, d and f) images of the droplets loaded with FITC-labeled latex particles with a diameter of 54 nm (a and b), 184 nm (c and d) and 1000 nm (e and f), all at ϕ_{NP} =0.0052. The scale bar is 100 μm. ϕ_0=0.043.

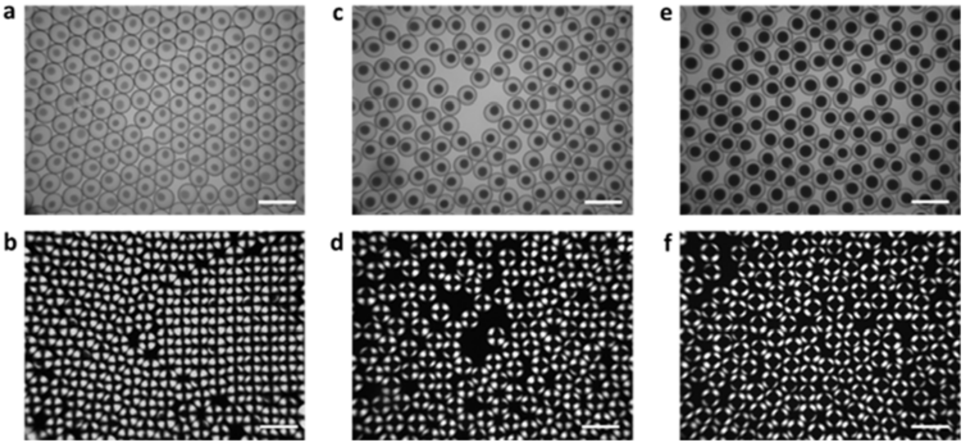

Supplementary Fig. 10. **Low-magnification images of droplets loaded with latex NPs at different NP volume fraction.** BF (a, c and e) and POM (b, d and f) images

of Ch-CNC droplets loaded with 184 nm FITC-labeled latex particles at ϕ_{NP} of 0.0022 (a and b), 0.0088 (c and d) and 0.0235 (e and f). The scale bar is 200 μm. ϕ_0 =0.043.

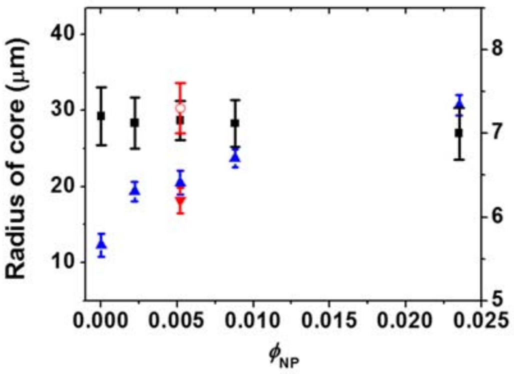

Supplementary Fig. 11. **Structural characteristics of droplets loaded with latex NPs.** Variation in the core radius (blue triangles) and pitch (black squares) of the Ch-CNC droplets loaded with 184 nm NPs. The red triangle and the red circle show the core radius and the pitch, respectively, for the droplets loaded with 54 nm latex NPs. ϕ_0=0.043.

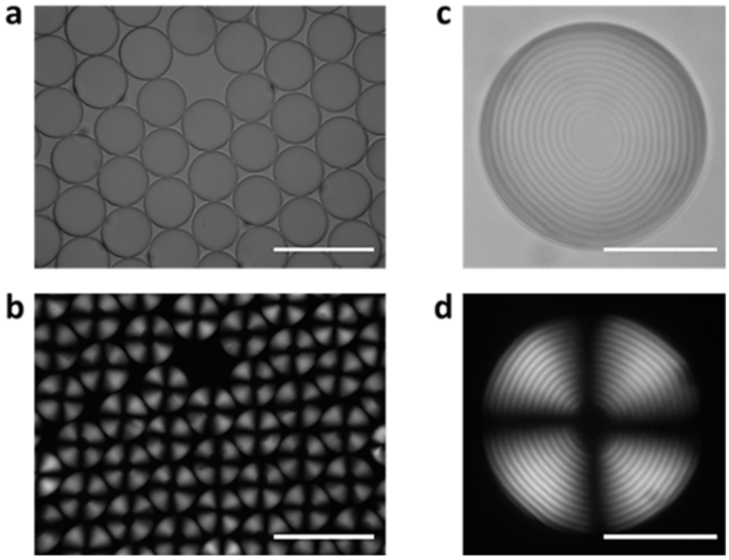

Supplementary Fig.12. **Ch-CNC droplets loaded with the supernatant of latex**

dispersion. BF (a and c) and POM (b and d) images of latex-free droplets prepared by mixing the Ch-CNC phase with 10 vol % of the supernatant of latex dispersion. The scale bar is 200 μm in (a, b), 50 μm in (c, d). ϕ_0=0.043.

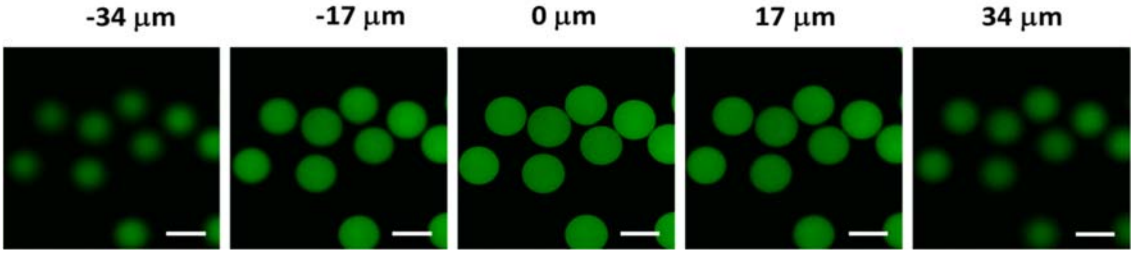

Supplementary Fig. 13. **Z-stack of FM images of the Ch-CNC droplets loaded with carbon dots.** The 0 μm position corresponds to the central plane of the droplets. The images were taken from the top (negative values) and the bottom (positive values) of the droplets. The concentration of carbon dots in the droplets is 1 mg mL^{-1}. The scale bar is 100 μm. ϕ_0=0.043.

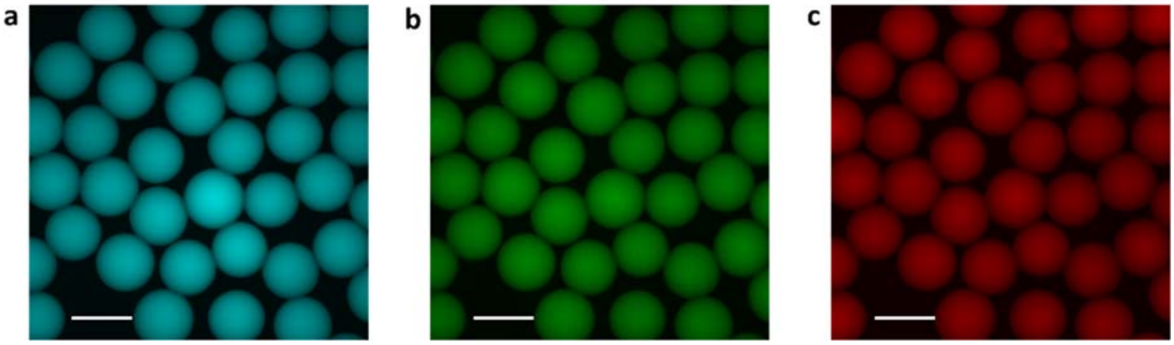

Supplementary Fig. 14. **Excitation dependent emission of droplets loaded with carbon dots.** FM images of the Ch-CNC droplets loaded with carbon dots at a concentration of 1 mg mL^{-1} and excited at λ_{exc} of 365 (a), 440 (b) and 550 nm (c). The scale bar is 100 μm. ϕ_0=0.043.

Supplementary Note 1

Phase separation in the macroscopic mixture of the Ch-CNC phase and latex NPs.

A mixture of the Ch-CNC phase (540 μL, ϕ_0=0.048) and the dispersion of 184 nm FITC-labeled latex NPs (60 μL, ϕ_{NP}=0.052) was shaken at 50 Hz for 3 min at 25 °C in a vortex mixer. The resultant volume fraction of latex NPs and CNCs in the mixed suspension was 0.0052and 0.043, respectively. The mixture was transferred into a cell consisting of two parallel glass slides separated with a 400 μm-thick spacer. The chamber was sealed with epoxy glue, and the phase separation was monitored using an inverted microscope (Nikon Eclipse-Ti). In a parallel experiment, addition, a mixture (500 μL) of the Ch-CNC phase and 184 nm latex NPs was introduced into a glass cylinder with a diameter of 0.6 cm. The volume fraction of CNCs in this mixed suspension was maintained at 0.043, while the volume fraction of the latex NPs was changed from 0 to 0.0235. Following equilibration for 18 days, the suspension phase-separated into a top latex-rich isotropic phase and a bottom a CNC-rich Ch phase. The images of these phases were acquired using a camera Nikon D7200. The heights of the two phases were determined by analyzing the images with a software ImageJ. The standard deviation in determining the heights of the isotropic and Ch phases did not exceed 0.1 mm.

In the mixture of the Ch-CNC phase and latex NPs, after 6 h equilibration, a large number of small latex-rich droplets formed (Supplementary Figure 6a and b), which subsequently, grew larger due to the time-dependent phase-separation and

coalescence.

Following equilibration for 18 days, the volume fraction of the latex-rich isotropic phase increased with increasing volume fraction of latex NPs in the mixture (Supplementary Fig. 6c).

Supplementary Note 2

Estimation of the free energy difference

To estimate the quantity $(f_i - f_{ch})$ in Eq. (1), we recast it through the difference in the pressures and chemical potentials of the two phases

$$f_i - f_{ch} = -(p_i - p_{ch}) + [\mu_i(p_i) - \mu_{ch}(p_{ch})] / v_{CNC} .$$

Then using expansion

$$\mu_i(p_i) \approx \mu_i(p_{ch}) + v_{CNC}(p_i - p_{ch}) + \ldots ,$$

one arrives at the estimate

$$f_i - f_{ch} = [\mu_i(p_{ch}) - \mu_{ch}(p_{ch})] / v_{CNC}$$

used in the main text.

Supplementary Methods

An aqueous CNC suspension with CNC concentration of 11.8 wt% was supplied by the Process Development Center of the University of Maine. The polydispersity of the CNC leght and diameter (183 and 23 nm, respectively) was 22 and 29 %, respectively, determined by analyzing their transmission electron

microscopy images. The electrokinetic potential of the CNCs was -47+/-9 mV (determined by dynamic light scattering). The photoresist SU-8 was purchased from MicroChem. Poly(dimethylsiloxane) (PDMS, Sylgard 184) was supplied by Dow Corning. The fluorescein isothiocyanate (FITC)-labeled polystyrene latex particles (Fluoresbrite® YG Carboxylate Microspheres) were purchased from Polysciences. The polydispersity of the latex particles with an average diameter of 54, 184 and 1000 nm was 10, 1.0, and 3.6 % respectively. The electrokinetic potential of the latex particles with an average diameter of 54, 184 or 1000 nm was -48+/-6, -41+/-6, and -37+/-17 mV respectively. Fluorinated oil HFE-7500 3M Novec was purchased from MG Chemicals. Thiol-terminated poly(ethylene glycol) (PEG-SH) with a molecular weight of M_n=5000 g/mol was purchased from Polymer Source Inc. The perfluoropolyether-co-poly(ethylene oxide-co-polypropylene oxide)-*co*-perfluoro-polyether copolymer [PFPE-P(EO-PO)-PFPE] was synthesized as described elsewhere[1]. All other chemicals were purchased from Aldrich Canada.

A CNC suspension (10 mL) with CNC concentration varying from 3 to 8 wt% (or volume fraction from 0.02 to 0.052.) was introduced into a glass vial with a diameter of 2.5 cm (The volume fraction of CNCs was calculated using the density of CNCs of 1.566 g/cm^3 [2,3]). The volume of an individual CNC, v_{CNC}, was calculated as $v_{CNC} = \pi \left(\frac{D}{2}\right)^2 L$, by assuming a cylindrical shape of CNCs. Following equilibration for 21 days, the suspension phase-separated into the top isotropic phase and the bottom cholesteric (Ch) phase. The images of these phases were acquired using a camera Nikon D7200. The heights of the phases were determined by analyzing the

images with a software ImageJ. The standard deviation in determining the heights of the isotropic and Ch phases did not exceed 0.2 mm. The volume fraction of the Ch phase increased with increasing volume fraction, ϕ_{CNC}, of CNCs in the original suspension (Supplementary Fig. 1a). Following phase separation, the Ch phase was removed from the vial with a syringe using a 22G needle. In the control experiment, the sealed vial with the separated Ch-CNC phase was equilibrated for 7 months. No further phase separation was observed.

Polarized optical microscopy (POM) images of the Ch phase were taken using an optical microscope (Olympus BX51) in the transmission mode for the Ch-CNC phase with CNC volume fraction ϕ_0=0.048 (Supplementary Fig. 1b and c). The multidomain mosaic pattern, with characteristic stripes clearly seen under high magnification, was consistent with the previous reports on the texture of the Ch-CNC phase[4–6]. In the control experiment, following phase separation of the original CNC suspension in the funnel, the bottom Ch-CNC phase was separated without using a syringe. The POM images of the Ch-CNC phases separated in these two experiments were qualitatively similar.

Gold nanoparticles (NPs) stabilized with oleylamine were synthesized using a procedure reported elsewhere[7]. As synthesized NPs dispersed in oleylamine were mixed with an excess of ethanol and precipitated by centrifugation at 3000 g for 5 min, followed by NP redispersion in chloroform, and subsequent purification from the excess of oleylamine by adding an excess of ethanol and centrifuging the dispersion at 3000 g for 5 min in 2 cycles. Then, 5 mL of chloroform solution containing 5 mg of

gold NPs was mixed with 5 mL of the solution of PEG-SH in chloroform (5 mg mL^{-1}). The mixture was stirred for 12 h at room temperature. The resulting dispersion was mixed with an equal volume of hexane and centrifuged at 3000 g for 5 min, followed by the redispersion in chloroform and two more cycles of purification *via* the addition of hexane and centrifugation. The resulting NPs were redispersed in 1 mL chloroform. After evaporation of chloroform, the remaining powder was dissolved in water, thus yielding a final 5 mL water solution of 10 nm-diameter gold NPs stabilized with PEG-SH (1 mg mL^{-1}). The average diameter of the NPs was 10 nm, the polydispersity was 20 %, and the electrokinetic potential was -23+/-9 mV.

Gold NPs with dimensions of 50 nm were prepared in a three-step procedure. First, gold seeds were prepared using a method reported elsewhere[8]. A solution of freshly prepared, ice-cold $NaBH_4$ in deionized water (10 mM, 0.6 mL) was added under stirring to a solution prepared by mixing aqueous cetyl trimethylammonium bromide (CTAB, 0.1 M, 9.833 mL) and $HAuCl_4$ (15 mM, 0.167 mL) in a 20 mL scintillation vial. After 2 min stirring, the pale yellow mixture was left undisturbed at room temperature for 2 h. This seed solution was diluted to 100 mL with deionized water. In another flask, growth solution was prepared by combining aqueous solutions of CTAB (0.24 M, 4 mL) with $HAuCl_4$ (15 mM, 0.133 mL) and ascorbic acid (0.1 M, 3 mL). The resulting clear solution was diluted to 50 mL with deionized water. To this solution, 0.6 mL of the diluted seed solution was added and the resulting mixture was shaken vigorously. The resultant pale pink-coloured solution was left undisturbed at room temperature for 30 min. Excess surfactant from this solution was removed by

using one cycle of centrifugation (20 000 g, 20 min), removing the supernatant and redispersing gold NP seeds with deionized water. To prepare 50 nm gold NPs, 4 mL of this purified seed solution was added to a 20 mL scintillation vial and diluted it to 10 mL with deionized water. Then, 1.6 mL of aqueous 0.1 M CTAB was added to this solution. The resultant mixed solution was heated to 30 ºC in a water bath for 5 min and 250 μL of 15 mM aqueous $HAuCl_4$ solution and 0.8 mL of 0.1 M ascorbic acid were added in succession. This solution was left undisturbed at room temperature for 15 min and was then concentrated to a 3 mL volume *via* centrifugation at 5000 g for 20 min. The concentrated solution was mixed with 5 mL of aqueous PEG-SH solution (5 mg mL^{-1}) and the mixture was stirred for 12 h. The resulting PEG-SH-stabilized gold NPs were purified from CTAB and excess of PEG-SH using 5 centrifugation cycles at 5000 g for 20 min, followed by redispersion in deionized water. The average diameter of gold NPs was 54 nm, polydispersity was 5%, and electrokinetic potential was -15+/-7 mV.

Carbon dots were synthesized following the procedure reported elsewhere[9]. Briefly, citric acid (1.0507 g) and ethylenediamine (335 μL) were dissolved in deionized water (10 mL). The solution was transferred to a poly(tetrafluoroethylene)-lined autoclave (30 mL) and heated at 200 ºC for 5 h. The reactor was cooled to room temperature. The brown-black transparent solution was subjected to dialysis by using dialysis bag with molecular weight cut-off of 10000 g mol^{-1} to obtain carbon dots in the solution outside of the dialysis bag. The average diameter of carbon dots was 3.5 nm, polydispersity was 15%, and electrokinetic potential was 2+/-5 mV.

The synthesis of Fe_3O_4@SiO_2 nanorods was carried out using a modified protocol reported previously[10]. In the first step, to synthesize FeOOH nanorods anhydrous $FeCl_3$ (8 g) was dissolved in 80 mL of water. This solution was added into 450 μL of 37 wt% HCl and the mixed solution was filtered using 220 nm pore size filter to remove insoluble precipitate. The resultant solution was heated for 16 h at 98 ºC in a 150 mL flask with refluxing. The yellow solid product (FeOOH nanorods) was collected and washed by using centrifugation (8000 g, 10 min). In the second step, the FeOOH nanorods were engulfed with a SiO_2 coating. The powder of FeOOH nanorods was dispersed in 20 mL of deionized water, and 0.1 mL 0.03 M poly(acrylic acid) solution was added into the nanorod dispersion. After overnight stirring, the nanorods were cleaned via centrifugation (8000 g, 10 min) and redispersion in 3 mL of deionized water. An ammonia solution (1 mL, 28 wt%) was added into the nanorod dispersion, followed by the addition of 20 mL ethanol and 100 μL of tetraethyl orthosilicate. After 1 h, the silica-coated nanorods collected by centrifugation, were washed several times by ethanol and redispersed in 2 mL of deionized water. In the third step, the FeOOH@SiO_2 nanorods were transformed into Fe_3O_4@SiO_2 nanorods. With the protection of the nitrogen atmosphere, 2 mL of the FeOOH@SiO_2 dispersion was added to 60 mL of diethylene glycol heated to 220 ºC. The color of the mixture changed from yellow to brownish, and finally, to black. The conversion of the FeOOH@SiO_2 nanorods into Fe_3O_4@SiO_2 nanorods took ~24 h. The resultant nanorods were collected via centrifugation, washed by excess of ethanol several times and re-dispersed in 5 mL of water. The resultant magnetic nanorods had an average

length and diameter of 1.1 μm and 100 nm, respectively. The polydispersity of the diameter and length of the nanorods were 18 and 24 %, respectively, and their electrokinetic potential was -26+/-5 mV.

Transmission electron microscopy (TEM) imaging of CNCs, latex NPs, gold NPs, carbon dots, and $Fe_3O_4@SiO_2$ nanorods was performed on a Hitachi H-7000 microscope. The extinction spectra of the solution of gold NPs were acquired using Cary-5000 spectrophotometer.

The microfluidic devices were fabricated in PDMS, using a standard soft lithography procedure[11]. Photoresist (SU-8) masters were prepared by photolithography on the silicon wafers. After fabrication, the microfluidic devices were maintained in an oven at 140 ºC for 12 h to increase hydrophobicity of the microchannel surface [12].

Supplementary References

1. Holtze, C. *et al.* Biocompatible surfactants for water-in-fluorocarbon emulsions. *Lab Chip* **8,** 1632–1639 (2008).
2. Lahiji, R. R. *et al.* Atomic force microscopy characterization of cellulose nanocrystals. *Langmuir* **26,** 4480–4488 (2010).
3. Pakzad, A., Simonsen, J., Heiden, P. A. & Yassar, R. S. Size effects on the nanomechanical properties of cellulose I nanocrystals. *J. Mater. Res.* **27,** 528–536 (2012).
4. Lagerwall, J. P. F. *et al.* Cellulose nanocrystal-based materials: from liquid

crystal self-assembly and glass formation to multifunctional thin films. *NPG Asia Mater.* **6,** e80 (2014).

5. Dong, X. M., Kimura, T., Revol, J.-F. & Gray, D. G. Effects of ionic strength on the isotropic - chiral nematic phase transition of suspensions of cellulose crystallites. *Langmuir* **12,** 2076–2082 (1996).

6. Moon, R. J., Martini, A., Nairn, J., Simonsen, J. & Youngblood, J. Cellulose nanomaterials review : structure , properties and nanocomposites. *Chem. Soc. Rev.* **40,** 3941–3994 (2011).

7. Lambright, S. *et al.* Enhanced lifetime of excitons in nonepitaxial Au / CdS core / shell. *ACS Nano* **8,** 352–361 (2014).

8. Fan, F. *et al.* Epitaxial growth of heterogeneous metal nanocrystals : from gold nano-octahedra to palladium and silver nanocubes. *J. Am. Chem.Soc.* **130,** 6949–6951 (2008).

9. Zhu, S. *et al.* Highly photoluminescent carbon dots for multicolor patterning , sensors , and bioimaging. *Angew. Chemie Int. Ed.* **52,** 3953–3957 (2013).

10. Wang, M., He, L., Zorba, S. & Yin, Y. Magnetically actuated liquid crystals. *Nano Lett.* **14,** 3966–3971 (2014).

11. Xia, Y. & Whitesides, G. M. Soft lithography. *Angew. Chemie Int. Ed.* **37,** 550–575 (1998).

12. Kumachev, A. *et al.* Biomaterials high-throughput generation of hydrogel microbeads with varying elasticity for cell encapsulation. *Biomaterials* **32,** 1477–1483 (2011).
